# An Epistatic Interaction between the *PAX8* and *STK17B* Genes in Papillary Thyroid Cancer Susceptibility

**DOI:** 10.1371/journal.pone.0074765

**Published:** 2013-09-23

**Authors:** Iñigo Landa, Cesar Boullosa, Lucía Inglada-Pérez, Ana Sastre-Perona, Susana Pastor, Antonia Velázquez, Veronika Mancikova, Sergio Ruiz-Llorente, Francesca Schiavi, Ricard Marcos, Nuria Malats, Giuseppe Opocher, Ramon Diaz-Uriarte, Pilar Santisteban, Alfonso Valencia, Mercedes Robledo

**Affiliations:** 1 Hereditary Endocrine Cancer Group, Spanish National Cancer Research Centre, Madrid, Spain; 2 Structural and Computational Biology Group, Spanish National Cancer Research Centre, Madrid, Spain; 3 Centro de Investigación Biomédica en Red de Enfermedades Raras, Madrid, Spain; 4 Instituto de Investigaciones Biomédicas Alberto Sols, Consejo Superior de Investigaciones Científicas, Autonomous University of Madrid, Madrid, Spain; 5 Departament de Genètica i de Microbiologia, Autonomous University of Barcelona, Barcelona, Spain; 6 Centro de Investigación Biomédica en Red de Epidemiologia y Salud Pública, Madrid, Spain; 7 Human Oncology and Pathogenesis Program, Memorial Sloan-Kettering Cancer Center, New York, New York, United States of America; 8 Familial Cancer Clinic and Oncoendocrinology, Veneto Institute of Oncology, Padova, Italy; 9 Genetic and Molecular Epidemiology Group, Spanish National Cancer Research Centre, Madrid, Spain; 10 Department of Medical and Surgical Sciences, University of Padova, Padova, Italy; 11 Departamento de Bioquímica, Universidad Autónoma de Madrid, Instituto de Investigaciones Biomédicas “Alberto Sols”, Consejo Superior de Investigaciones Científicas, Madrid, Spain; MOE Key Laboratory of Environment and Health, School of Public Health, Tongji Medical College, Huazhong University of Science and Technology, China

## Abstract

Papillary Thyroid Cancer (PTC) is a heterogeneous and complex disease; susceptibility to PTC is influenced by the joint effects of multiple common, low-penetrance genes, although relatively few have been identified to date. Here we applied a rigorous combined approach to assess both the individual and epistatic contributions of genetic factors to PTC susceptibility, based on one of the largest series of thyroid cancer cases described to date. In addition to identifying the involvement of *TSHR* variation in classic PTC, our pioneer study of epistasis revealed a significant interaction between variants in *STK17B* and *PAX8*. The interaction was detected by MD-MBR (p = 0.00010) and confirmed by other methods, and then replicated in a second independent series of patients (MD-MBR p = 0.017). Furthermore, we demonstrated an inverse correlation between expression of *PAX8* and *STK17B* in a set of cell lines derived from human thyroid carcinomas. Overall, our work sheds additional light on the genetic basis of thyroid cancer susceptibility, and suggests a new direction for the exploration of the inherited genetic contribution to disease using association studies.

## Introduction

Follicular-cell-derived thyroid carcinomas are the most common endocrine malignancies and their incidence has remarkably increased in recent years [1], [2]. Among them, papillary thyroid carcinoma (PTC, 80–85% of cases), and follicular thyroid carcinoma (FTC, 5–10%) are the most frequent subtypes [3]. It is widely accepted that follicular-cell-derived thyroid cancer behaves as a complex disease, where multiple genetic variants, located on low-penetrance genes (LPG), interact with each other and with the environment, thus modulating individual susceptibility [4]–[6].

We previously reported the association of the *FOXE1* gene with PTC susceptibility [7], a result that has been extensively replicated [8]–[10]. Until now, most association studies have focused on main effects and identified only single, or a very limited number of, genes involved in PTC pathogenesis. Variants in these genes explain a relatively small proportion of cases. While additional low-penetrance genes may be identified by future studies of main effects, it is also likely that common variants at different *loci* interact to modify susceptibility. When genetic variants affect the phenotype jointly in a non-additive way, this gene-gene interaction is known as epistasis. The detection of epistatic interactions represents not only statistical, but also computational challenges [11], [12]. These can be overcome by focusing association studies on genes that *a priori* could play a role together, either because they are located on the same pathways, or because they are differentially expressed in thyroid tumors. The relative risk of thyroid cancer for first degree relatives of probands is higher than that for any other non-Mendelian neoplasia [13]–[15], suggesting a stronger genetic component to its etiology and therefore a possibly higher likelihood of identifying gene-gene interactions.

The aim of this study was to obtain a more comprehensive view of the genetic basis of PTC by, on the one hand further assessing the implication of common variants in candidate genes, and on the other testing for epistatic two-way interactions between these variants. To this end, a two-step association approach was adopted, based on one of the largest differentiated thyroid cancer patient series described so far. It included a discovery set of 609 cases and 525 controls (series I), and two independent replication series, comprising 969 cases and 1040 controls (series II and III). We identified and replicated an interaction between variants in *PAX8* and *STK17B*, suggesting they may be new players in thyroid cancer susceptibility. Functional assays confirmed that the expression of these genes is inversely correlated, although the underlying mechanism leading to the development of cancer has still to be elucidated.

## Results

### Expanded Replication Series Further Confirm the Involvement of *FOXE1* in PTC Susceptibility

We further independently replicated the previously reported association with common variation in the *FOXE1* gene [7]. In our second, more recently collected Spanish case-control series (series III), comprising 451 PTC cases and 540 controls, we confirmed the highly significant association for functional variant rs1867277 in the *FOXE1* promoter region, under the same multiplicative model, with an OR (per allele) of 1.44 (95%CI = 1.19–1.74; *P* = 2.0×10^−4^). Overall, based on a combined total of 1358 PTC cases and 1551 controls of white European origin from Spain and Italy, a per-allele OR of 1.45 was estimated (95%CI = 1.30–1.61; P = 4.7×10^−12^).

### Stratification of Patients Unveils Putative Subtype-specific Associations between Individual SNPs and Thyroid Cancer Risk

After genotyping cases and controls in the discovery stage, we selected 9 variants located on 9 different genes for inclusion in the replication stage. Each SNP was either the most significant tagSNP at a given *locus*, or was consistently predicted to be functional, as shown in Table 1.

**Table 1:** Association with risk of follicular cell-derived thyroid cancer for 9 candidate variants in the discovery and replication stages.

SNP (alleles)^a^	Gene	Location, function	Group of cases	Best fitting model	Discovery^d^			Replication^e^		
					MAF in cont; cases	OR (95% CI)^b^	*P* c	MAF in cont; cases	OR (95% CI)^b^	*P* c
rs16973034 (A/G)	*PRKAR1A*	intronic, tagSNP	PTC	dominant	0.153; 0.108	0.63 (0.47–0.84)	1.8×10^−3^	0.138; 0.133	1.00 (0.79–1.26)	0.984
rs2703488 (T/C)	*KIT*	intronic, tagSNP	PTC	dominant	0.488; 0.551	1.43 (1.07–1.91)	1.5×10^−2^	0.476; 0.472	0.93 (0.75–1.16)	0.522
rs4939827 (T/C)	*SMAD7*	intronic, tagSNP	PTC	recessive	0.461; 0.416	0.68 (0.50–0.94)	1.9×10^−2^	0.438; 0.478	1.32 (1.03–1.68)	0.027
rs2066807 (G/C)	*STAT2*	exonic, p.Met594Ile	PTC	dominant	0.033; 0.054	1.69 (1.06–2.69)	2.6×10^−2^	0.040; 0.039	0.98 (0.68–1.42)	0.928
rs2284734 (A/G)	*TSHR*	intronic, tagSNP	cPTC	recessive	0.290; 0.388	2.64 (1.69–4.13)	1.8×10^−5^	0.304; 0.317	1.42 (0.99–2.03)	0.058
rs1053266 (G/T)	*CCDC6*	exonic, p.Pro470Thr	cPTC	recessive	0.494; 0.543	1.37 (0.96–1.97)	8.7×10^−2^	0.471; 0.502	1.24 (0.96–1.60)	0.103
rs2687834 (G/T)	*TG*	intronic, tagSNP	fvPTC	recessive	0.447; 0.565	2.28 (1.50–3.46)	1.1×10^−4^	0.491; 0.477	0.74 (0.46–1.17)	0.195
rs6179 (G/A)	*GHR*	exonic, ESE	FTC	dominant	0.356; 0.232	0.46 (0.27–0.80)	5.4×10^−3^	0.299; 0.286	0.87 (0.55–1.37)	0.543
rs13099828 (C/G)	*PPARG*	intronic, tagSNP	fvPTC+FTC	dominant	0.186; 0.256	1.71 (1.22–2.38)	1.6×10^−3^	0.168; 0.189	1.01 (0.71–1.46)	0.936

Abbreviations: MAF = minor allele frequency; OR = odds ratio; CI = confidence interval; ESE = Exonic Splicing Enhancers; PTC = Papillary Thyroid Carcinoma; cPTC = classic PTC; fvPTC = follicular variant of PTC; FTC = Follicular Thyroid Carcinoma. The table is sorted by disease subtype and, within each group, by *P*-value.

a Major/minor allele (in controls);

b OR and CI were obtained using homozygotes for the most frequent allele in controls as the reference group;

c *P*-values are derived from Wald statistics;

d Results adjusted for age and gender;

e Results adjusted for age, gender and country.

The most significant association in the discovery stage was obtained for SNP rs2284734 in the *TSHR* gene (recessive OR = 2.64; 95% CI = 1.69–4.13; *P* = 1.8×10^−5^), suggesting its involvement as a risk factor specific to the development of classic PTC. We also observed evidence of a subtype-specific effect for rs2687834 in the *TG* gene (recessive OR = 2.28; 95% CI = 1.50–3.46; *P* = 1.1×10^−4^), further suggesting that tumor stratification appears to be a relevant aspect to consider in association studies. This is shown graphically in Figure S1. The association of *TSHR*-rs2284734 with cPTC was marginally statistically significant in the replication stage (recessive OR = 1.42; *P* = 0.058, Table 1). The associations for remaining 8 SNPs were not replicated.

### Epistatic Interaction between *STK17B* and *PAX8* in Thyroid Cancer Susceptibility

Among all PTC cases, one interaction was consistently observed in analyses by three methods; epistasis between the SNP-pair rs721992 and rs6554198, located in the *CCDC6* and *KIT* genes respectively, was detected by MDR, SNPHarvester and MB-MDR (Table 2). However, this interaction was not replicated in series II and III (p = 0.13).

**Table 2:** Table 2. Gene-gene interaction results for susceptibility to Papillary Thyroid Carcinoma overall.

SNP1	GENE	SNP2	GENE	MDR	SH	MECPM	MSH	MBMDR*
rs721992	***CCDC6***	rs6554198	***KIT***					**<0.0001**
rs11985450	***ChGn***	rs3021526	***FOXE1***					0.0637
rs2395911	***SLC26A4***	rs1799977	***MLH1***					0.0002
rs16945753	***MED13***	rs2979033	***TG***					0.0045
rs8069645	***STAT3***	rs2979040	***TG***					0.0035
rs10119760	***FOXE1***	rs2958681	***TG***					0.0190
rs3111800	***KIT***	rs4607021	***ITGB2***					0.0010
rs10119760	***FOXE1***	rs15866	***STK17A***					0.0084
rs6556301	***FGFR4***	rs6948512	***SHH***					0.0085
rs2958681	***TG***	rs7350420	***NCOA4***					0.0298
rs1044217	***STK17A***	rs2958681	***TG***					0.0114
rs12667481	***SLC26A4***	rs6180	***GHR***					NA
rs440555	***ITGB2***	rs6180	***GHR***					0.0332
rs16945753	***MED13***	rs17202345	***CCDC6***					0.0735
rs1648305	***DUOX1***	rs3098233	***CTHRC1***					0.0854
rs2703488	***KIT***	rs6018257	***SRC***					0.0785
rs17705719	***TG***	rs15997	***RAF1***					0.1085

SH- SNPHarvester; MSH- MegaSNPHunter; light grey shading indicates interactions selected by each of the algorithms; dark grey shading indicates the interaction fulfilling the established cutoff (SNP pair selected by at least three methods);

* associated P-value; NA- not available (all genotypes combinations classified as neutral).

**Figure 1:**
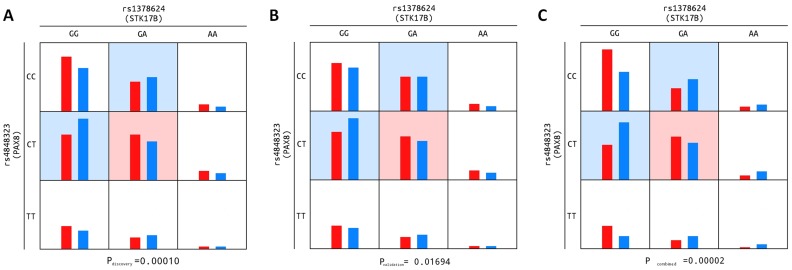
Epistatic model for SNPs in *PAX8* and *STK17B* and genotype frequencies for cPTC-cases and controls. Relative frequencies of the nine genotype combinations of the replicated interaction (*PAX8*-*STK17B*) are shown for cases and controls (red and blue columns, respectively). The cell containing the high-risk genotype combination is highlighted in light red, those with low-risk combinations in light blue, and those with neutral combinations are uncoloured. Figure1a - based on the discovery stage (series I); Figure 1b - based on the replication stage (series II and III); Figure 1c – based on both stages combined (series I, II and III).

When only considering patients diagnosed with cPTC, one interaction was detected by four out of the five methods applied. This interaction, involving SNPs rs4848323 and rs1378624, located in the *PAX8* and *STK17B* genes, respectively, had an associated p-value by MB-MDR of 0.00010 in the discovery stage and of 0.017 in the replication phase (Table 3, Figure 1). The corresponding p-value from the combined analysis of data from both stages was 0.00002, estimated by 100,000 permutations. This interaction was consistently observed by MB-MDR when the entire PTC case-series (including all subtypes) was considered (p-value = 0.026 and 0.045 for the discovery and replication stages, respectively).

**Table 3:** Gene-gene interaction results for susceptibility to classic Papillary Thyroid Carcinoma.

SNP1	GENE	SNP2	GENE	MDR	SH	MECPM	MSH	MBMDR*
rs4848323	***PAX8***	rs1378624	***STK17B***					**<0.0001**
rs3758249	***FOXE1***	rs1017141	***TSHR***					NA
rs1465618	***THADA***	rs1041457	***ITGB2***					0.0003
rs2979040	***TG***	rs9912773	***STAT3***					0.0005
rs10755938	***TG***	rs1706804	***DUOX1***					0.0632
rs2229642	***ITPR3***	rs4128209	***THADA***					0.0005
rs16945753	***MED13***	rs4682845	***PTHR1***					0.0027
rs10974947	***JAK2***	rs1051340	***TRIP11***					0.0045
rs12691874	***CXCR4***	rs4128209	***THADA***					0.0482
rs12941827	***MED13***	rs2411256	***FGFR1***					0.0011
rs3763743	***CCDC6***	rs2025488	***CGA***					0.0858
rs11985450	***ChGn***	rs4927632	***TPO***					0.0789
rs17786733	***TPO***	rs2017472	***KIT***					0.0016
rs1998008	***TSHB***	rs6018257	***SRC***					0.0409
rs11972418	***SLC26A4***	rs901854	***ITPR1***					0.0024
rs2284735	***TSHR***	rs27438	***CSF2***					0.0052
rs1568400	***THRA***	rs2298566	***SNX19***					0.0033
rs180202	***TG***	rs10940495	***IL6ST***					0.0974
rs2701684	***SLC26A4***	rs6948512	***SHH***					0.0083
rs1466018	***PAX8***	rs6554198	***KIT***					0.0232
rs7559891	***THADA***	rs881301	***FGFR1***					0.0234
rs1799977	***MLH1***	rs7048394	***FOXE1***					0.0033
rs1624715	***CCDC6***	rs12949918	***STAT3***					0.0198
rs26279	***MSH3***	rs920964	***CCDC6***					NA
rs10146516	***TSHR***	rs2464196	***TCF1***					0.049
rs11030043	***RHOG***	rs2838738	***ITGB2***					0.0006
rs11903287	***THADA***	rs7581626	***STK17B***					NA
rs172310	***SHH***	rs6018257	***SRC***					NA
rs2734871	***CXCR4***	rs16945753	***MED13***					0.0284
rs2395911	***SLC26A4***	rs310247	***JAK1***					NS
rs1799977	***MLH1***	rs17413525	***THADA***					0.0017
rs11535853	***TG***	rs4939827	***SMAD7***					0.0772
rs2235978	***TRIP11***	rs6873545	***GHR***					0.0551

SH- SNPHarvester; MSH- MegaSNPHunter; Light grey shading indicates interactions selected by each of the algorithms; dark grey green shading indicates the interaction fulfilling the established criteria to pass to stage 2 (replication) (SNP pair selected by at least three methods);

* associated P-value; NA- not available (all genotypes combinations classified as neutral).

**Figure 2:**
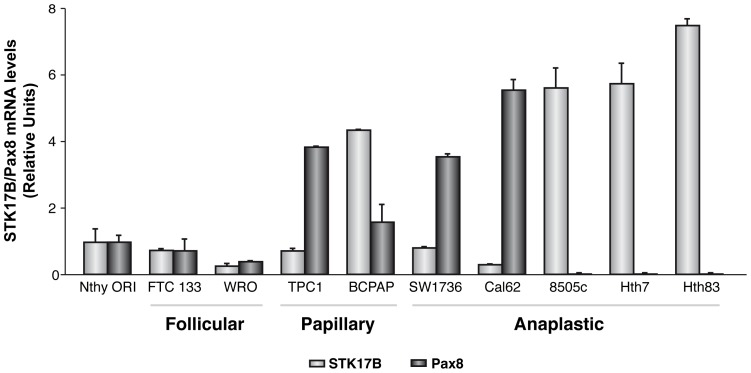
STK17B and PAX8 mRNA levels in thyroid tumoral cell lines. The mRNA expression levels of both genes were determined by qRT-PCR and are represented as relative units. The name of the cell line and its origin (follicular, papillary or anaplastic) is indicated.

In order to gain functional insights into the PAX8-STK17B interaction, we first assessed the expression of these genes using data from a previous array-based study of 63 thyroid tumors [16] and observed an inverse correlation (r = −0.77; p = 8.65×10^−14^; Figure S2). We subsequently observed a consistent result in a series of thyroid cancer cell lines from different human tumors; high STK17B expression was observed in the most undifferentiated human anaplastic thyroid cells (8505c, Hth7 and Hth83), which are characterized by very low or null PAX8 levels (Figure 2).

**Figure 3:**
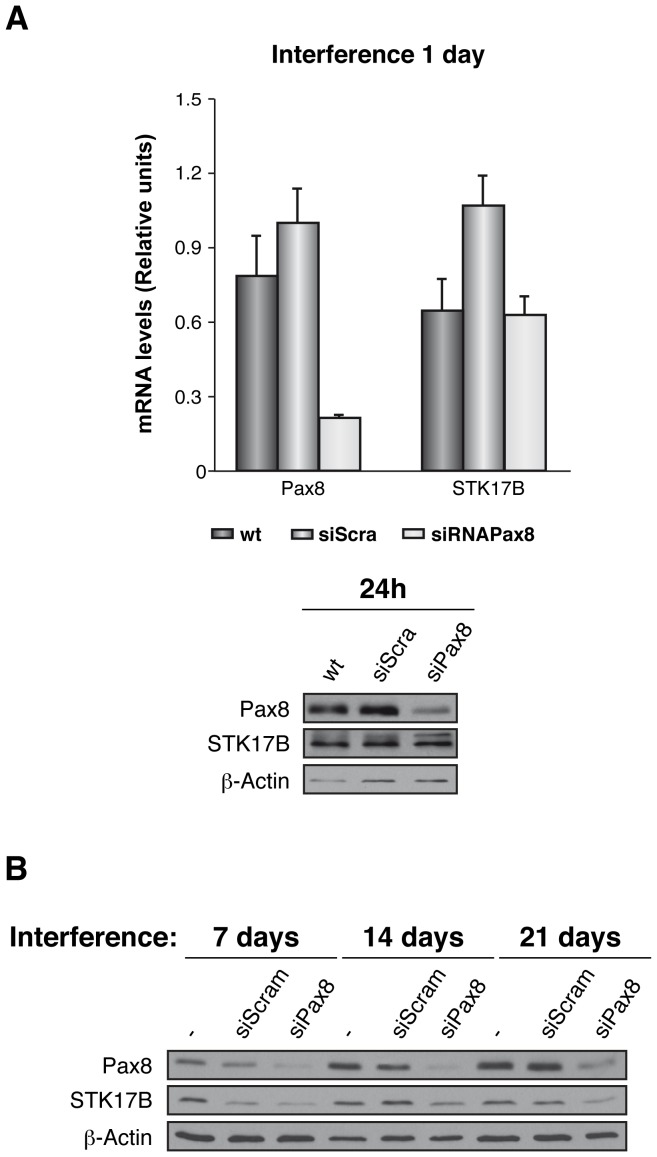
Relative expression levels of STK17B in Pax8-silenced cells. PCCl3 cells were transiently (A) or stably (B) silenced for the transcription factor Pax8 (siPax8). As a control, wild type or siScramble transfected cells were used. The expression levels were assessed by means of qRT-PCR (A, upper panel) or western blot (A, lower panel, and B).

To explore the relationship between PAX8 and STK17B in thyroid cancer, rat thyroid cells were Pax8-silenced and the STK17B expression analyzed. After one day of transient silencing of PAX8, mRNA levels of PAX8 decreased by approximately 70% relative to the wild type or the siScamble transfected cells. However, no change was observed in the mRNA levels of STK17B. Pax8 protein levels were also lower in the silenced cells while STK17b levels remained unchanged relative to the siScamble transfected cells (Fig. 3A). To determine whether the absence of a correlation was due to the short-term nature of the silencing, a siPax8-stable cell line was generated and the protein levels assayed 7, 14 and 21 days after transfection. Again, a decrease in PAX8 levels was found without a substantial variation in STK17B levels. (Fig. 3B).

## Discussion

Although the number of identified LPGs for follicular-cell-derived thyroid carcinoma has increased in recent years, it still lags behind that for other complex diseases. As recently reviewed, associations have been consistently replicated for only a limited number of *loci* [4], [6], thus a large proportion of the heritability of this multifactorial disease remains unexplained.

Thyroid cancer has a strong genetic component, with the relative risk for first-degree relatives of probands being the highest among neoplasias not displaying regular Mendelian inheritance [13]–[15]. A focus on the identification of new genes through the assessment of gene-gene interaction may therefore unveil at least part of this unexplained genetic component. In recent years, the large number of susceptibility variants identified for complex diseases has given rise to the need to perform specific analyses to evaluate their potential epistatic effects. The importance of these analyses lies mainly in unveiling how the presence of one genetic variant influences the effect of another variant [17]–[19].

Such epistatic analyses, unlike those of genetic main effects, constitute a computational and statistical challenge. Many methods have been developed to detect epistasis, but all of them have limitations and their performance is variable. In addition, some of these methods are computationally unviable for GWAS data [20]. Since our study considered only 768 SNPs in genes chosen on the basis of their *a priori* putative role in the disease, it was possible to apply several more complex algorithms derived from complementary strategies, thus increasing the robustness of interaction detection.

The use of MB-MDR in the second replication stage implied several advantages, including the possibility to test the exact epistatic model identified in the discovery stage, and the ability to adjust for potential confounders, as well as marginal SNP effects, the latter to avoid the influence on the results of SNP pairs acting in purely additive way. Furthermore, a permutation-based p-value could be readily obtained at a reasonable computational cost. The fact that we were able to replicate one of the two interactions detected in the discovery stage is testament to the reliability of our proposed pipeline.

To date, a multitude of studies performed with yeast models have identified unequivocal gene-gene interactions [21]–[24]. On the contrary, attempts to identify epistatic or other kind of interactions in human disease susceptibility have only relatively recently started to yield results [25]–[29]. Nevertheless, few if any have been convincingly replicated. The present study has, for the first time, identified and independently replicated an epistatic association with cPTC susceptibility of common variants in two genes. This success is likely due not only to having a reduced list of candidate genes, but also to the high heritability of thyroid cancer, as well as the careful clinical characterization of the case series to identify disease subtypes with minimal error.

Our analysis of epistasis, applied to a case-control study based on a candidate gene approach [7], has identified an interaction between *PAX8* and *STK17B*. This and future findings of epistatic genetic associations could be the first step towards a deeper biological characterization of thyroid cancer. *STK17B* encodes DRAK2, a serine-threonine kinase involved in the regulation of apoptosis. It was initially selected as a candidate gene to study based on its differential expression in PTC tumors relative to normal thyroid tissue [7], [16]. *PAX8* was chosen because it is a well-known thyroid transcription factor, related to thyroid differentiation, the regulation of specific genes, and to congenital diseases [30]–[33]. Interestingly, a recent study combining a genome-wide analysis of Pax8 binding sites with gene expression profiles has defined apoptosis as an important pathway under Pax8 regulation [34]. Although the functional assays performed by siRNA did not explain the underlying biological mechanism, gene expression studies based on both human tumors and cell lines have found an inverse correlation between *PAX8* and *STK17B*. This latter finding corroborates the findings from the association study, suggesting that these two *loci* indeed interact to influence susceptibility to cPTC. Thus, it is tempting to speculate that PAX8 might play a role in the regulation of *STK17B* expression, pointing to this latter, relatively unknown gene as a new putative player in thyroid metabolism.

Finally, our results for individual variants in *TSHR* and possibly *TG*, and the epistatic effect of the SNPs in *PAX8* and *STK17B*, all apparently stronger for specific disease subtypes, stress the potential importance of tumor characterisation and stratification in association studies. The association of tagSNP rs2284734 in *TSHR* with risk of cPTC was the second most statistically significant (OR = 2.64; 95% CI = 1.69–4.13; *P* = 1.8×10^−5^), with consistent results observed in the replication stage (OR = 1.42; 95% CI = 0.99–2.03; *P* = 0.058). Several studies have suggested an association between *TSHR* and susceptibility of developing autoimmune pathologies of the thyroid gland [35]–[38], one of them involving the same tagSNP (rs2284734). An association with Graves’ disease has also been reported [39]. Few studies have assessed the association between *TSHR* and thyroid cancer risk and those that have, have reported negative results so far [40], [41]. However, previous studies have evaluated only a limited number of exonic *TSHR* variants. It seems premature to rule out this *locus* in thyroid cancer susceptibility. Fine-mapping of the region should be performed in order to identify a functional variant, possibly intronic and/or regulatory, to explain our observed association with cPTC.

In summary, we propose additional genetic factors that may explain part of the unresolved heritability of thyroid cancer. In addition to identifying a potential role of *TSHR* variants in cPTC risk, we have detected and independently replicated for the first time an epistatic relationship between the *PAX8* and *STK17B* genes in thyroid cancer susceptibility. This gene-gene interaction demonstrates that epistatic effects may play an important role on thyroid cancer, and could explain the lack of an observed association for each gene individually. Further studies are needed to determine whether gene-gene interactions can be useful as risk markers for follicular cell-derived thyroid cancers, as well as for other tumors.

## Materials and Methods

### Ethics Statement

Written informed consent was obtained from all participants in accordance protocols approved by the “Comité de bioética y bienestar animal del Instituto de Salud Carlos III” and the Ethics Committee of the COR (Regional Cancer Center), Padova, Italy, which approved this study.

### Subjects

Three series of thyroid cancer cases and controls were recruited, as described below:

#### Discovery (series I)

We recruited 609 thyroid cancer patients from the Spanish hospital network. These included the main thyroid follicular-cell derived carcinomas: 520 PTC, represented by the main subtypes ‘classic PTC’ (cPTC; n = 304) and ‘follicular variant PTC’ (fvPTC; n = 146); and 69 follicular thyroid carcinomas (FTC). Medullary thyroid carcinomas (MTC) were not included in the study.

A series of 525 cancer-free controls were recruited from the same geographical regions covered by the hospitals involved in the study. For both case and controls the mean age was 46 years and the female:male sex ratio was 4.6∶1.

#### Replication (series II and III combined)

A second case-control series (series II) comprised 412 PTC and 44 FTC patients recruited at three hospitals located in Italy and 500 controls from the same three geographical regions.

A third, independent group of Spanish thyroid cancer patients were obtained, including 451 PTC and 62 FTC, as well as a complementary set of 540 Spanish controls (series III).

Overall, the replication study comprised 969 thyroid cancer cases of white European ancestry, including 863 PTC (582 cPTC and 118 fvPTC ) and 106 FTC. Their mean age was 47 years and the female:male sex ratio was 4.4∶1. The mean age of the total 1040 controls was 53 years and the female:male sex ratio was 2.4∶1.

Clinicians from all participating centers completed a detailed clinical questionnaire for each patient that included personal and clinical information, such as tumor subtype and stage, as well as details of surgery, treatment and the development of metastasis during follow-up.

### DNA Isolation and Quantification

Blood or saliva samples were obtained from all cases and controls. Genomic DNA was extracted from peripheral blood lymphocytes by automated methods according to the manufacturer’s instructions (Magnapure, Roche, Madrid, Spain), or manually, using standard methods [42]. DNA was extracted from saliva using the Oragene DNA Self-Collection Kit (DNA Genotek, Ottawa, Canada). DNA concentration was quantified in all samples prior to genotyping using the Quant-iT PicoGreen dsDNA Reagent (Invitrogen, Eugene, OR, USA).

### Gene and SNP Selection

Ninety-seven candidate genes were selected in a biologically oriented manner and 768 single nucleotide polymorphisms (SNPs) (Table S1) were identified therein, as previously described [7].

### SNP Genotyping

SNPs were genotyped in the discovery stage using the Illumina GoldenGate® Genotyping Assay (San Diego, CA, USA) system, on a Sentrix Universal-96 Array Matrix multi-sample array format. Genotyping for the replication stage was carried out using the KASPar SNP Genotyping System (Kbiosciences, Herts, UK) as previously described [7].

### Statistical Analysis

Departure from Hardy-Weinberg equilibrium (HWE) for all SNPs was tested in controls using Fisher’s exact test. SNP main effects were assessed by estimating genotype-specific odds ratios (OR) via unconditional logistic regression, using homozygotes of the most frequent allele in controls as the reference group. For each SNP, the best fitting genetic model was determined, and the corresponding p-value was calculated based on the Wald statistic. All models were adjusted for the putative confounding factors age, gender, and when relevant, country. We assessed heterogeneity in the per-allele OR by thyroid cancer subtype using a likelihood-ratio test, as previously described [43]. Statistical analyses were performed using SPSS for Windows 17.0 and STATA version 10®, unless otherwise stated.

### Methods to Assess SNP-SNP Interactions

We tested for epistasis using cases from homogeneous and robustly represented disease groups, including PTC overall and cPTC. We assessed two-way interactions between SNPs using five different methods, Multifactor Dimensionality Reduction (MDR) [44], [45], Maximum Entropy Conditional Probability Modeling (MECPM) [46], SNPHarvester [47], MegaSNPHunter [48], and Model Based - Multifactor Dimensionality Reduction (MB-MDR) [49]; see Methods S1). We adjusted for sex and age in the discovery stage, where permitted by the method in question. Only interactions (SNP pairs) identified by at least three methods were selected for replication in series II and III. We used MB-MDR to test in the replication stage the epistatic model identified in the discovery stage (see Methods S1). Age, sex and country were included as covariates. MB-MDR assigns each of the nine possible combined two-SNP genotypes to three risk categories (high, low, and neutral) using logistic regression. We included age, sex and country as covariates and computed p-values based on a permutation test [44], [45]. Replication of interactions was assessed by forcing the genotype combinations into the risk categories determined in the discovery stage. Interactions with an associated p-value <0.05 were considered replicated. A similar strategy was applied to combined data from both stages for replicated interactions.

### Functional Assays

#### Cell culture

Cell lines were kindly donated by Dr. Heldin (SW1736, Hth7 and Hth83 [50]), Dr. Fagin (WRO [51]) and Dr. Fusco and Dr. Santoro (BCPAP [52] and TPC-1 [53]), or obtained from the German Collection of Microorganism and Cells Culture (Cal62, 8505c) and the European collection of cell culture (NthyORI, FTC133). All cell lines were genetically fingerprinted and verified to be unique and of thyroid origin [54].

PCCl3 cells are a continuous line of rat thyroid follicular cells that express the thyroid-specific transcription factors Nkx2.1, Foxe1, and Pax8. They were grown in Coon’s modification of Ham’s F-12 medium, supplemented with 5% donor calf serum and a six-hormone mixture [55].

The human cell lines used were derived from normal thyroid tissue or from follicular, papillary or anaplastic thyroid carcinomas. 8505c, WRO and SW1736 cells, as well as NthyORI3.1 control cells were grown in RPMI medium, while FTC133, TPC1, BCPAP and Cal62 cells were growth in DMEM medium, and Hth7 and Hth83 cells in MEM medium. All the media were supplemented with 5% fetal bovine serum.

#### Generation of PCCl3-Pax8-silenced thyroid cells

The silencing of Pax8 was performed in PCCl3 cells either transiently or stably. Transient transfection was carried out using the DharmaFECT 1 siRNA transfection reagent both for scrambled and for *Pax8* siRNA conditions (Dharmacon, Denver, CO). Stable short hairpin RNA (shRNA) was obtained by infection with pGIPZ lentiviral plasmid containing the interfering sequence of puromycin-resistant Pax8 or its control (scramble) (Open Biosystem, Denver, CO).

Total RNA was obtained from control (wild type) and from transiently-Pax8-silenced cells (si*Pax8* or scrambled siRNA) using the TRIzol reagent (Invitrogen, Carlsbad, CA) following the manufacturer’s recommended protocol; semiquantitative RT-PCR was then performed as previously described [56] using specific primers for Pax8 (*forward* CAAGGTGGTGGAGAAGATTG and *reverse* GAGGTTGAATGGTTGCTG), STK17B (*forward* CCTGAGTTGGCTGAAATG and *reverse* TCTGTTGCTGTGGTAATGGG) or βactin (*forward* CACTCTTCCAGCCTTCCTT and *reverse* CTCGTCATACTCCTGCTTGCT).

Validation of Pax8 silencing and STK17B expression was tested either by RT-PCR or by Western blot using a polyclonal Pax8 mouse antibody (Biopat, Milan, Italy) or a human STK17B antibody (RD System, Minneapolis, MN), respectively. βactin levels were used as loading control after immunoblotting with a specific antibody (Santa Cruz Biotechnology, CA).

## Supporting Information

Figure S1 **Subtype-specific Manhattan plot representations of the differences in allelic frequencies between cases and controls in the discovery series.** Upper panel: classic PTC *vs.* controls; Lower panel: follicular variant of PTC *vs.* controls. Highlighted areas correspond to the variants of *TSHR* and *TG*, specifically associated to each of the mentioned PTC subtypes, respectively. The inserted table shows the results for the top two variants in their specific subtypes, as well as the correspondent *P*-values derived from the likelihood-ratio test, thus demonstrating the subtype-specificity of *TSHR* and *TG* associations.
(TIF)

Figure S2 **Unsupervised clustering for PAX8 and STK17B probes in our previously published mRNA array, including 63 thyroid tumors (Montero-Conde et al, 2008– ref. 26).** A significant inverse correlation is observed (r = −0.77; p = 8.65×10^−14^). Abbreviations: PTC = Papillary Thyroid Carcinoma; FVPTC = follicular variant of PTC; FTC = Follicular Thyroid Carcinoma; FA = Follicular Adenoma; PDTC = Poorly Differentiated Thyroid Carcinoma; ATC = Anaplastic Thyroid Carcinoma; N = Normal Thyroid.
(TIF)

Table S1 **Complete list of SNPs studied in the discovery series.**
(DOC)

Methods S1 **Brief description of the methods used to detect epistatic interactions.**
(DOC)
