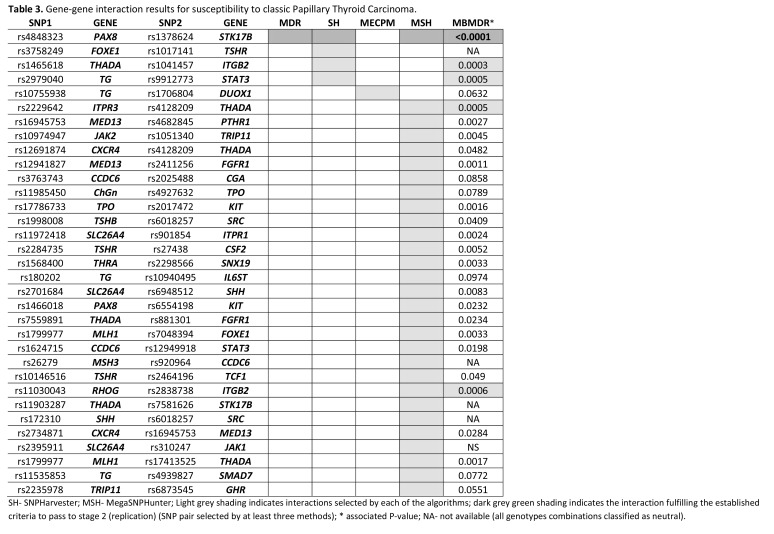# Correction: An Epistatic Interaction between the *PAX8* and *STK17B* Genes in Papillary Thyroid Cancer Susceptibility

**DOI:** 10.1371/annotation/cd94c3eb-70f2-4dfa-85be-b3fc41e495c3

**Published:** 2013-10-21

**Authors:** Iñigo Landa, Cesar Boullosa, Lucía Inglada-Pérez, Ana Sastre-Perona, Susana Pastor, Antonia Velázquez, Veronika Mancikova, Sergio Ruiz-Llorente, Francesca Schiavi, Ricard Marcos, Nuria Malats, Giuseppe Opocher, Ramon Diaz-Uriarte, Pilar Santisteban, Alfonso Valencia, Mercedes Robledo

There were errors in Tables 2 and 3. The correct versions of these tables are available below.

Figure 2: 

**Figure:**
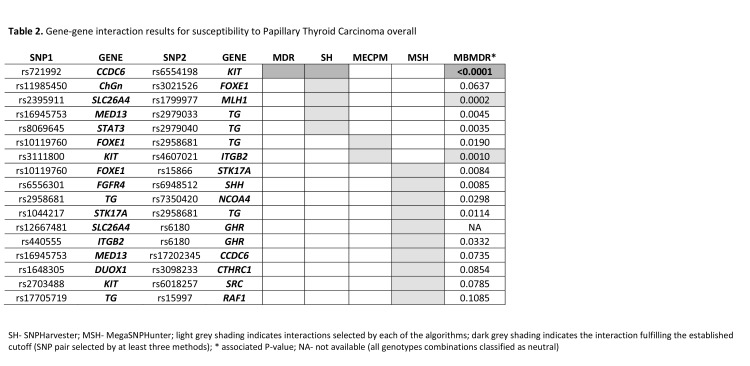


Figure 3: 

**Figure:**