# Supplementary figures and images for: An Epistatic Interaction between the PAX8 and STK17B Genes in Papillary Thyroid Cancer Susceptibility
Source: PLoS One. 2013 Sep 23;8(9):e74765. doi: 10.1371/journal.pone.0074765 (PMC3781145; doi:10.1371/journal.pone.0074765)

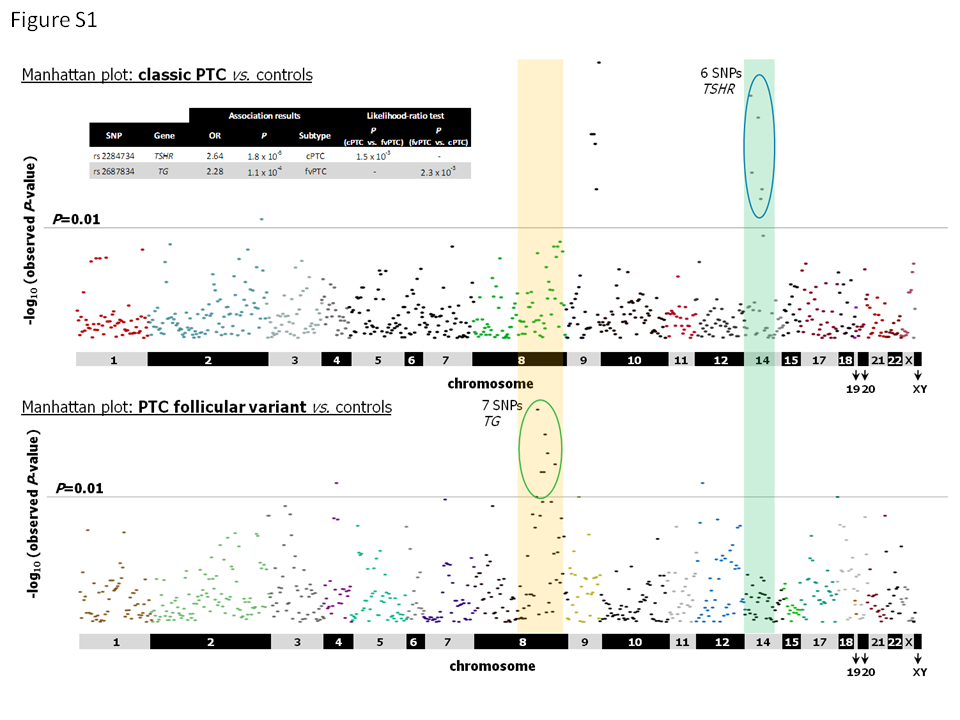

Supplement: Figure S1 — Subtype-specific Manhattan plot representations of the differences in allelic frequencies between cases and controls in the discovery series. Upper panel: classic PTC vs. controls; Lower panel: follicular variant of PTC vs. controls. Highlighted areas correspond to the variants of TSHR and TG, specifically associated to each of the mentioned PTC subtypes, respectively. The inserted table shows the results for the top two variants in their specific subtypes, as well as the correspondent P-values derived from the likelihood-ratio test, thus demonstrating the subtype-specificity of TSHR and TG associations. (TIF) [file pone.0074765.s001.tif]

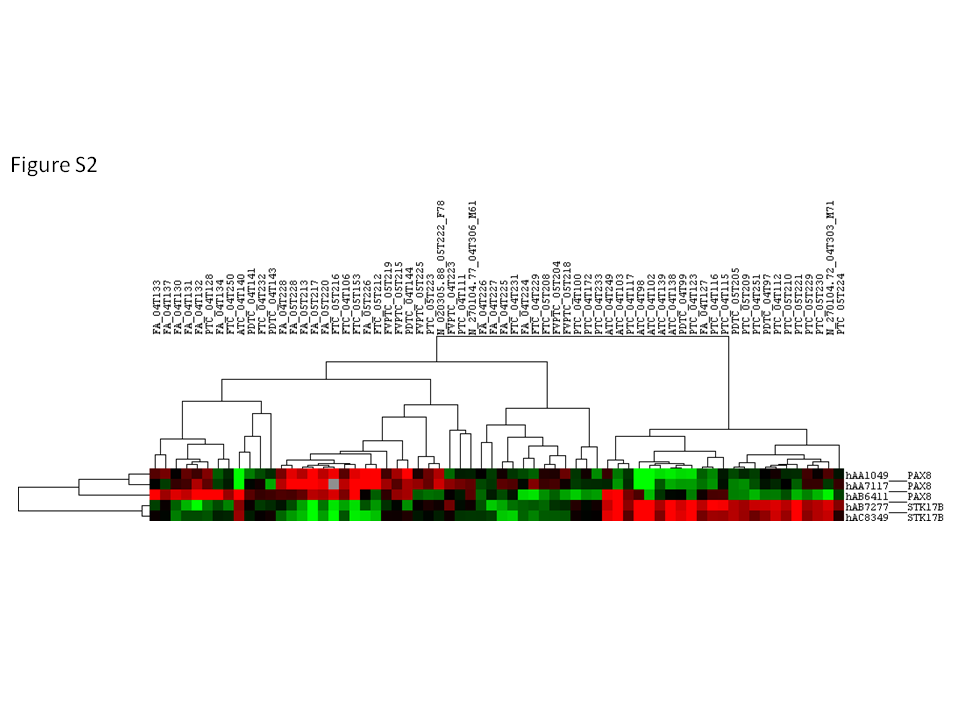

Supplement: Figure S2 — Unsupervised clustering for PAX8 and STK17B probes in our previously published mRNA array, including 63 thyroid tumors (Montero-Conde et al, 2008– ref. 26). A significant inverse correlation is observed (r = −0.77; p = 8.65×10^−14^). Abbreviations: PTC = Papillary Thyroid Carcinoma; FVPTC = follicular variant of PTC; FTC = Follicular Thyroid Carcinoma; FA = Follicular Adenoma; PDTC = Poorly Differentiated Thyroid Carcinoma; ATC = Anaplastic Thyroid Carcinoma; N = Normal Thyroid. (TIF) [file pone.0074765.s002.tif]
