# Supplementary material for: An Epistatic Interaction between the PAX8 and STK17B Genes in Papillary Thyroid Cancer Susceptibility
Source: PLoS One. 2013 Sep 23;8(9):e74765. doi: 10.1371/journal.pone.0074765 (PMC3781145; doi:10.1371/journal.pone.0074765)
Supplement: Table S1 — Complete list of SNPs studied in the discovery series. (DOC) [file pone.0074765.s003.doc]

**Table S1.** Complete list of SNPs studied in the discovery series.

| 1_23935574_rs10917420_tag_TCEB3_b1 |
| --- |
| 1_23944942_rs550850_tag_TCEB3_b1 |
| 1_23948463_rs2294495_tag_TCEB3_b1 |
| 1_23950038_rs2235541_non.syn_TCEB3_b1 |
| 1_23956236_rs2076346_tag_TCEB3_b1 |
| 1_54138952_rs12131461_tag_DIO1_b1 |
| 1_54148289_rs11206244_ESE_DIO1_r1 |
| 1_54153994_rs12063777_tag_DIO1_b2 |
| 1_65075926_rs310241_tag_JAK1_b3 |
| 1_65076098_rs4916004_tag_JAK1_b3 |
| 1_65076247_rs12129819_tag_JAK1_b3 |
| 1_65077058_rs11579616_tag_JAK1_b3 |
| 1_65078465_rs310244_tag_JAK1_b3 |
| 1_65079997_rs310247_tag_JAK1_b3 |
| 1_65083077_rs2230588_ESE_JAK1_b4 |
| 1_65083802_rs3737139_tag_JAK1_b4 |
| 1_65083850_rs2230587_tag_JAK1_b4 |
| 1_65084930_rs17127063_ESE_JAK1_b4 |
| 1_65093838_rs11579263_ESE_JAK1_b4 |
| 1_65106445_rs310236_tag_JAK1_b5 |
| 1_65108419_rs17127114_tag_JAK1_b5 |
| 1_65115093_rs12135754_tag_JAK1_b5 |
| 1_65118204_rs310209_tag_JAK1_b5 |
| 1_65130866_rs17390852_TFBS_JAK1_r1 |
| 1_115366523_rs11102872_TFBS_TSHB_b1 |
| 1_115367714_rs7515454_TFBS_TSHB_b1 |
| 1_115367826_rs7517739_TFBS_TSHB_b1 |
| 1_115368615_rs12040783_tag_TSHB_b2 |
| 1_115369408_rs1575070_TFBS_TSHB_b2 |
| 1_115369551_rs1575069_tag_TSHB_b2 |
| 1_115373439_rs1321109_tag_TSHB_b2 |
| 1_115377238_rs11102875_tag_TSHB_b2 |
| 1_115377546_rs10776792_non.syn_TSHB_b2 |
| 1_115382063_rs1998008_tag_TSHB_b2 |
| 1_115387924_rs4611011_tag_TSHB_b2 |
| 1_149056488_rs10305741_non.syn_ARNT_r1 |
| 1_150212253_rs3007700_tag_S100A10_b1 |
| 1_150212403_rs16833728_tag_S100A10_b1 |
| 1_150218187_rs12128371_tag_S100A10_b1 |
| 1_150218319_rs2999526_tag_S100A10_b1 |
| 1_150221633_rs6678672_tag_S100A10_b1 |
| 1_150228199_rs11204922_tag_S100A10_b1 |
| 1_150233369_rs1038745_TFBS_S100A10_b1 |
| 1_155041296_rs2644609_tag_NTRK1_b1 |
| 1_155043341_rs928391_tag_NTRK1_b1 |
| 1_155052241_rs1800601_tag_NTRK1_b2 |
| 1_155053552_rs2768764_tag_NTRK1_b2 |
| 1_155059756_rs7517728_tag_NTRK1_b3 |
| 1_155061630_rs4661222_tag_NTRK1_b3 |
| 1_155063468_rs6427332_tag_NTRK1_b3 |
| 1_155069281_rs4661061_tag_NTRK1_b3 |
| 1_155075830_rs4661229_tag_NTRK1_b3 |
| 1_155091425_rs4661063_TFBS_NTRK1_r1 |
| 1_155101171_rs6340_ESE_NTRK1_r2 |
| 1_155112857_rs6334_ESE_NTRK1_r3 |
| 1_155115542_rs6336_non.syn_NTRK1_r4 |
| 1_155115619_rs6337_ESE_NTRK1_r5 |
| 1_234448448_rs1055851_non.syn_ERO1LB_r1 |
| 2_1388360_rs9677074_tag_TPO_b1 |
| 2_1389936_rs17786733_TFBS_TPO_b1 |
| 2_1393966_rs2298873_TFBS_TPO_b1 |
| 2_1470181_rs7574221_tag_TPO_b10 |
| 2_1475429_rs11687944_tag_TPO_b10 |
| 2_1478934_rs732609_non.syn_TPO_b10 |
| 2_1481670_rs3755552_tag_TPO_b10 |
| 2_1487692_rs4927621_tag_TPO_b10 |
| 2_1493291_rs13414566_tag_TPO_b10 |
| 2_1499683_rs1126799_non.syn_TPO_r1 |
| 2_1519491_rs10181280_tag_TPO_b13 |
| 2_1524117_rs4927632_tag_TPO_b13 |
| 2_1525334_rs1042589_non.syn_TPO_b13 |
| 2_43400339_rs9808360_tag_THADA_b5 |
| 2_43401176_rs17334247_non.syn_THADA_b5 |
| 2_43401632_rs17030684_tag_THADA_b5 |
| 2_43407453_rs1465618_tag_THADA_b5 |
| 2_43408233_rs4128209_tag_THADA_b5 |
| 2_43410988_rs17406174_tag_THADA_b5 |
| 2_43469512_rs6739828_tag_THADA_b9 |
| 2_43472323_rs11899863_tag_THADA_b9 |
| 2_43472544_rs10166054_tag_THADA_b9 |
| 2_43478582_rs7605661_tag_THADA_b9 |
| 2_43481559_rs7600177_tag_THADA_b9 |
| 2_43483726_rs17030749_tag_THADA_b9 |
| 2_43483963_rs17030751_TFBS_THADA_b9 |
| 2_43530443_rs12105399_tag_THADA_b16 |
| 2_43539241_rs6738365_tag_THADA_b16 |
| 2_43539711_rs2046916_tag_THADA_b16 |
| 2_43562870_rs999949_tag_THADA_b16 |
| 2_43564570_rs4146253_tag_THADA_b16 |
| 2_43571154_rs6746064_tag_THADA_b16 |
| 2_43586327_rs7578597_non.syn_THADA_b16 |
| 2_43591677_rs1038822_tag_THADA_b16 |
| 2_43608496_rs1491508_tag_THADA_b16 |
| 2_43620543_rs7559891_tag_THADA_b17 |
| 2_43630357_rs6742108_tag_THADA_b17 |
| 2_43631158_rs11903287_tag_THADA_b17 |
| 2_43633169_rs1873555_tag_THADA_b17 |
| 2_43639779_rs17413525_tag_THADA_b17 |
| 2_43651123_rs17031056_non.syn_THADA_b17 |
| 2_43655216_rs11899823_ESE_THADA_b17 |
| 2_43683044_rs10182489_tag_THADA_b17 |
| 2_95319407_rs2278067_tag_KCNIP3_b1 |
| 2_95323539_rs889854_tag_KCNIP3_b1 |
| 2_95326275_rs3755525_tag_KCNIP3_b1 |
| 2_95329773_rs3772044_tag_KCNIP3_b1 |
| 2_95352576_rs2113418_tag_KCNIP3_b1 |
| 2_95387358_rs3755518_tag_KCNIP3_b1 |
| 2_95387391_rs3821340_tag_KCNIP3_b1 |
| 2_95398530_rs6712299_tag_KCNIP3_b3 |
| 2_95420738_rs2320433_tag_KCNIP3_b3 |
| 2_113690667_rs874898_tag_PAX8_b2 |
| 2_113691537_rs1478_ESE_PAX8_b2 |
| 2_113695411_rs11123170_tag_PAX8_b3 |
| 2_113700974_rs2241975_tag_PAX8_b3 |
| 2_113705707_rs2863242_tag_PAX8_b3 |
| 2_113717336_rs2863244_tag_PAX8_b4 |
| 2_113718391_rs13007173_tag_PAX8_b4 |
| 2_113720374_rs1491584_tag_PAX8_b4 |
| 2_113733499_rs6734610_tag_PAX8_b5 |
| 2_113735060_rs895417_tag_PAX8_b5 |
| 2_113737318_rs1466018_tag_PAX8_b5 |
| 2_113739039_rs6542126_tag_PAX8_b5 |
| 2_113741872_rs1446246_tag_PAX8_b5 |
| 2_113749237_rs4848323_tag_PAX8_b5 |
| 2_113758446_rs17626434_tag_PAX8_b6 |
| 2_113763028_rs11123176_tag_PAX8_b6 |
| 2_136584543_rs13022389_tag_CXCR4_b2 |
| 2_136586343_rs2734871_tag_CXCR4_b2 |
| 2_136589554_rs2228014_ESE_CXCR4_b2 |
| 2_136595086_rs9973445_tag_CXCR4_b2 |
| 2_136596944_rs12691874_tag_CXCR4_b2 |
| 2_136598575_rs6751768_tag_CXCR4_b2 |
| 2_160516321_rs3828323_non.syn_PLA2R1_r1 |
| 2_160593688_rs3749117_non.syn_PLA2R1_r2 |
| 2_196701858_rs12469216_tag_STK17B_b1 |
| 2_196709152_rs7581626_tag_STK17B_b1 |
| 2_196719027_rs16845711_tag_STK17B_b1 |
| 2_196753322_rs1378624_tag_STK17B_b1 |
| 2_215944957_rs17449032_non.syn_FN1_r1 |
| 2_227368788_rs1801278_non.syn_IRS1_r1 |
| 2_227369287_rs1801123_ESE_IRS1_r2 |
| 2_227370997_rs2234931_ESE_IRS1_r3 |
| 2_230324789_rs7565973_tag_TRIP12_b1 |
| 2_230331371_rs7571826_tag_TRIP12_b1 |
| 2_230333546_rs6758201_tag_TRIP12_b1 |
| 2_230337382_rs1044822_tag_TRIP12_b1 |
| 2_230338935_rs11696097_tag_TRIP12_b1 |
| 2_230340512_rs6687_ESE_TRIP12_b1 |
| 2_230393214_rs488007_tag_TRIP12_b1 |
| 2_230432021_rs544480_ESE_TRIP12_b1 |
| 2_230472470_rs17258702_tag_TRIP12_b1 |
| 2_230479357_rs831372_tag_TRIP12_b1 |
| 2_230495305_rs12993164_TFBS_TRIP12_b1 |
| 3_4691811_rs2306877_ESE_ITPR1_r1 |
| 3_4817231_rs2291862_ESE_ITPR1_r2 |
| 3_4831180_rs711631_ESE_ITPR1_r3 |
| 3_4831234_rs901854_ESE_ITPR1_r4 |
| 3_12309416_rs2972164_tag_PPARG_b1 |
| 3_12319441_rs7620165_tag_PPARG_b1 |
| 3_12334049_rs2067819_tag_PPARG_b1 |
| 3_12347386_rs4145573_tag_PPARG_b1 |
| 3_12363563_rs10510418_tag_PPARG_b1 |
| 3_12368125_rs1801282_non.syn_PPARG_b1 |
| 3_12377474_rs1373641_tag_PPARG_b2 |
| 3_12388339_rs2120825_tag_PPARG_b2 |
| 3_12389420_rs6809832_tag_PPARG_b2 |
| 3_12399793_rs2972162_tag_PPARG_b2 |
| 3_12412237_rs4135268_tag_PPARG_b2 |
| 3_12418844_rs4135275_tag_PPARG_b2 |
| 3_12437024_rs709157_tag_PPARG_b2 |
| 3_12441214_rs1175542_tag_PPARG_b2 |
| 3_12443589_rs13099828_tag_PPARG_b3 |
| 3_12445239_rs1797912_tag_PPARG_b3 |
| 3_12450088_rs7626560_tag_PPARG_b3 |
| 3_12450557_rs3856806_ESE_PPARG_r1 |
| 3_12599070_rs15997_tag_RAF1_b1 |
| 3_12599763_rs7956_tag_RAF1_b1 |
| 3_12600747_rs1051208_ESE_RAF1_b1 |
| 3_12601394_rs3730296_ESE_RAF1_b1 |
| 3_12601516_rs3729931_tag_RAF1_b1 |
| 3_12619335_rs1039244_tag_RAF1_b2 |
| 3_12621255_rs6777175_TFBS_RAF1_b2 |
| 3_12631016_rs4234512_tag_RAF1_b2 |
| 3_12634494_rs4684867_tag_RAF1_b2 |
| 3_12637370_rs11705805_tag_RAF1_b2 |
| 3_12638835_rs11923427_tag_RAF1_b2 |
| 3_12680048_rs5746154_tag_RAF1_b2 |
| 3_37028572_rs1799977_non.syn_MLH_r1 |
| 3_46900543_rs11926707_tag_PTHR1_b1 |
| 3_46904550_rs6442037_tag_PTHR1_b1 |
| 3_46906482_rs4683301_tag_PTHR1_b2 |
| 3_46910531_rs724450_tag_PTHR1_b2 |
| 3_46916120_rs2242116_tag_PTHR1_b2 |
| 3_46923530_rs7652849_tag_PTHR1_b2 |
| 3_46926939_rs11719795_tag_PTHR1_b2 |
| 3_46930408_rs4682845_tag_PTHR1_b2 |
| 3_151941695_rs8403_ESE_SIAH2_r1 |
| 3_151970635_rs2014279_TFBS_SIAH2_r2 |
| 4_55216917_rs6554198_tag_KIT_b2 |
| 4_55219490_rs3822213_tag_KIT_b2 |
| 4_55220687_rs2237037_tag_KIT_b2 |
| 4_55221008_rs2237035_tag_KIT_b2 |
| 4_55223410_rs2237034_tag_KIT_b2 |
| 4_55226632_rs2237032_tag_KIT_b2 |
| 4_55230093_rs2237029_tag_KIT_b3 |
| 4_55231132_rs2237028_tag_KIT_b3 |
| 4_55232238_rs759083_tag_KIT_b3 |
| 4_55236636_rs2237025_tag_KIT_b4 |
| 4_55247087_rs13135792_tag_KIT_b4 |
| 4_55249088_rs2703488_tag_KIT_b4 |
| 4_55266198_rs3111800_tag_KIT_b4 |
| 4_55283293_rs2298976_tag_KIT_b5 |
| 4_55283476_rs11735550_tag_KIT_b5 |
| 4_55285378_rs4864920_tag_KIT_b5 |
| 4_55294193_rs1008658_tag_KIT_b7 |
| 4_55294413_rs1124007_tag_KIT_b7 |
| 4_55296940_rs12643468_tag_KIT_b7 |
| 4_55297522_rs3733542_tag_KIT_b7 |
| 4_55299697_rs17084733_ESE_KIT_b7 |
| 4_55301967_rs3796776_tag_KIT_b7 |
| 4_55304790_rs4356963_tag_KIT_b7 |
| 4_141702921_rs2270565_non.syn_UCP_r1 |
| 5_42659309_rs6887528_tag_GHR_b5 |
| 5_42659702_rs7727694_tag_GHR_b5 |
| 5_42667021_rs6873545_tag_GHR_b5 |
| 5_42715853_rs4547964_tag_GHR_b5 |
| 5_42735801_rs6179_ESE_GHR_b5 |
| 5_42753367_rs12187996_tag_GHR_b5 |
| 5_42754996_rs6180_non.syn_GHR_b5 |
| 5_42755583_rs2910875_ESE_GHR_b5 |
| 5_42760670_rs1364024_tag_GHR_b5 |
| 5_52237479_rs4145748_non.syn_ITGA_r1 |
| 5_52250338_rs2279587_non.syn_ITGA_r2 |
| 5_52265502_rs12520591_non.syn_ITGA_r3 |
| 5_55259686_rs13357543_tag_IL6ST_b1 |
| 5_55272131_rs10471960_tag_IL6ST_b1 |
| 5_55275763_rs1900173_tag_IL6ST_b1 |
| 5_55279795_rs11574780_tag_IL6ST_b1 |
| 5_55280534_rs7719246_tag_IL6ST_b1 |
| 5_55287688_rs2228043_non.syn_IL6ST_b1 |
| 5_55298417_rs10940495_tag_IL6ST_b1 |
| 5_55336283_rs6450361_TFBS_IL6ST_r1 |
| 5_80204693_rs26279_non.syn_MSH3_r2 |
| 5_89979327_rs2366777_non.syn_MASS1_r1 |
| 5_90015274_rs17544552_non.syn_MASS1_r2 |
| 5_90015506_rs16868972_non.syn_MASS1_r4 |
| 5_90021638_rs10037067_non.syn_MASS1_r5 |
| 5_90024260_rs2366926_non.syn_MASS1_r6 |
| 5_90035966_rs16869016_non.syn_MASS1_r8 |
| 5_90052627_rs16869032_non.syn_MASS1_r9 |
| 5_90142864_rs2438374_non.syn_MASS1_r11 |
| 5_90187345_rs2247870_non.syn_MASS1_r12 |
| 5_131429914_rs11575022_TFBS_CSF2_r1 |
| 5_131430637_rs3091338_tag_CSF2_b1 |
| 5_131431992_rs11242103_tag_CSF2_b1 |
| 5_131432345_rs31473_tag_CSF2_b1 |
| 5_131434943_rs25879_tag_CSF2_b1 |
| 5_131435392_rs4705916_TFBS_CSF2_b1 |
| 5_131439037_rs25881_tag_CSF2_b1 |
| 5_131439359_rs25882_non.syn_CSF2_b2 |
| 5_131441154_rs27438_tag_CSF2_b2 |
| 5_176445853_rs640180_tag_FGFR4_b1 |
| 5_176450067_rs446382_ESE_FGFR4_b1 |
| 5_176450403_rs376618_non.syn_FGFR4_b1 |
| 5_176452849_rs351855_non.syn_FGFR4_b1 |
| 5_176460183_rs6556301_tag_FGFR4_b1 |
| 5_176464378_rs7708357_tag_FGFR4_b1 |
| 6_4661015_rs3812179_non.syn_CDYL_r1 |
| 6_33767450_rs2229642_non.syn_ITPR3_r1 |
| 6_87845883_rs17449571_tag_CGA_b1 |
| 6_87850120_rs9362387_tag_CGA_b1 |
| 6_87851299_rs9359730_tag_CGA_b1 |
| 6_87851978_rs6631_tag_CGA_b1 |
| 6_87852029_rs1055409_ESE_CGA_b2 |
| 6_87852582_rs7745823_tag_CGA_b2 |
| 6_87854623_rs6155_ESE_CGA_b2 |
| 6_87860673_rs6924434_tag_CGA_b2 |
| 6_87862004_rs9344675_TFBS_CGA_b2 |
| 6_87865149_rs1998616_TFBS_CGA_b2 |
| 6_87868159_rs17450398_TFBS_CGA_b2 |
| 6_87870842_rs6909901_tag_CGA_b3 |
| 6_87870921_rs2025488_TFBS_CGA_b3 |
| 7_5993133_rs1805318_non.syn_PMS2_r1 |
| 7_43589331_rs10251937_ESE_STK17A_b1 |
| 7_43602694_rs10271892_tag_STK17A_b1 |
| 7_43611958_rs7802995_tag_STK17A_b1 |
| 7_43620734_rs10259269_tag_STK17A_b2 |
| 7_43624282_rs12702053_tag_STK17A_b2 |
| 7_43625329_rs4077509_tag_STK17A_b3 |
| 7_43625707_rs6950861_tag_STK17A_b3 |
| 7_43626289_rs4077435_tag_STK17A_b3 |
| 7_43627418_rs1053080_tag_STK17A_b3 |
| 7_43629948_rs3779062_non.syn_STK17A_b3 |
| 7_43630805_rs1044141_non.syn_STK17A_b3 |
| 7_43631002_rs15866_tag_STK17A_b3 |
| 7_43631310_rs1044217_ESE_STK17A_b3 |
| 7_43637757_rs2330918_tag_STK17A_b3 |
| 7_45899194_rs4619_non.syn_IGFBP_r1 |
| 7_100328733_rs1799805_non.syn_ACHE_r1 |
| 7_100329387_rs17885778_non.syn_ACHE_r2 |
| 7_107081387_rs2712230_tag_SLC26A4_b1 |
| 7_107086763_rs2701684_tag_SLC26A4_b1 |
| 7_107087576_rs2712228_tag_SLC26A4_b1 |
| 7_107090864_rs2248465_tag_SLC26A4_b1 |
| 7_107095577_rs6949189_tag_SLC26A4_b2 |
| 7_107101183_rs11769313_tag_SLC26A4_b2 |
| 7_107104411_rs10276013_tag_SLC26A4_b2 |
| 7_107108438_rs10250105_tag_SLC26A4_b2 |
| 7_107115551_rs1858929_tag_SLC26A4_b4 |
| 7_107121262_rs2237679_tag_SLC26A4_b5 |
| 7_107137863_rs17154353_non.syn_SLC26A4_b5 |
| 7_107146620_rs12667481_tag_SLC26A4_b5 |
| 7_107153087_rs11972418_tag_SLC26A4_b5 |
| 7_128633564_rs2228617_tag_SMO_b1 |
| 7_128638111_rs2016607_ESE_SMO_b1 |
| 7_128639617_rs2075780_ESE_SMO_b1 |
| 7_128640089_rs3824_ESE_SMO_b1 |
| 7_128640256_rs1061275_ESE_SMO_b1 |
| 7_128640428_rs1061282_ESE_SMO_b1 |
| 7_128640544_rs1061285_ESE_SMO_b1 |
| 7_155297702_rs9333594_ESE_SHH_r1 |
| 7_155299433_rs288746_tag_SHH_b1 |
| 7_155305818_rs6948512_tag_SHH_b1 |
| 7_155308388_rs172310_tag_SHH_b1 |
| 8_19307283_rs3802330_ESE_ChGn_r1 |
| 8_19389160_rs11204047_tag_ChGn_b9 |
| 8_19396413_rs17479581_tag_ChGn_b9 |
| 8_19402576_rs4484715_tag_ChGn_b9 |
| 8_19407048_rs7017776_non.syn_ChGn_b9 |
| 8_19407705_rs6984644_ESE_ChGn_b9 |
| 8_19493906_rs7841402_tag_ChGn_b17 |
| 8_19500550_rs4922066_tag_ChGn_b17 |
| 8_19500753_rs4244454_non.syn_ChGn_b17 |
| 8_19584012_rs6985674_tag_ChGn_b25 |
| 8_19585246_rs4548199_tag_ChGn_b25 |
| 8_19587601_rs1994789_tag_ChGn_b25 |
| 8_19588068_rs17481221_TFBS_ChGn_b25 |
| 8_19591563_rs4922086_tag_ChGn_b25 |
| 8_38405382_rs2288696_tag_FGFR1_b1 |
| 8_38406370_rs2915665_ESE_FGFR1_b1 |
| 8_38408060_rs2978083_tag_FGFR1_b1 |
| 8_38412051_rs2978076_tag_FGFR1_b1 |
| 8_38418872_rs6987534_tag_FGFR1_b1 |
| 8_38441503_rs6996321_tag_FGFR1_b2 |
| 8_38444952_rs2467531_ESE_FGFR1_b2 |
| 8_38451252_rs7829058_tag_FGFR1_b2 |
| 8_38451475_rs881301_tag_FGFR1_b2 |
| 8_42155734_rs1136159_non.syn_PLAT_r1 |
| 8_42297491_rs17875749_non.syn_IKBKB_r1 |
| 8_42307589_rs17611716_non.syn_IKBKB_r2 |
| 8_99012774_rs2290472_non.syn_MATN2_r1 |
| 8_99060397_rs1869609_non.syn_MATN2_r2 |
| 8_99113704_rs2255317_non.syn_MATN2_r3 |
| 8_104443583_rs10096207_TFBS_CTHRC1_b1 |
| 8_104443839_rs10109642_tag_CTHRC1_b1 |
| 8_104444492_rs827595_tag_CTHRC1_b1 |
| 8_104445801_rs827598_TFBS_CTHRC1_b1 |
| 8_104446089_rs827599_tag_CTHRC1_b1 |
| 8_104454892_rs2575695_tag_CTHRC1_b1 |
| 8_104463920_rs3098233_ESE_CTHRC1_b1 |
| 8_120033233_rs2073618_non.syn_TNFRSF11B_r1 |
| 8_133938444_rs1916273_tag_TG_b1 |
| 8_133939006_rs2467993_TFBS_TG_b1 |
| 8_133946112_rs17683567_tag_TG_b1 |
| 8_133946609_rs180194_TFBS_TG_b1 |
| 8_133949394_rs180202_tag_TG_b1 |
| 8_133950426_rs180204_tag_TG_b1 |
| 8_133968342_rs180222_non.syn_TG_b1 |
| 8_133969434_rs180223_non.syn_TG_b1 |
| 8_133969568_rs2069550_ESE_TG_b1 |
| 8_133975788_rs10435562_tag_TG_b1 |
| 8_133979156_rs853326_non.syn_TG_b1 |
| 8_133986770_rs2068128_tag_TG_b2 |
| 8_133989700_rs2069556_non.syn_TG_b2 |
| 8_133991009_rs2687834_tag_TG_b2 |
| 8_133995875_rs2687820_tag_TG_b2 |
| 8_134000365_rs853305_tag_TG_b2 |
| 8_134000930_rs853304_ESE_TG_b2 |
| 8_134028366_rs2205452_tag_TG_b2 |
| 8_134041895_rs2142384_tag_TG_b3 |
| 8_134044465_rs2069561_non.syn_TG_b3 |
| 8_134050928_rs17693031_ESE_TG_r1 |
| 8_134052230_rs10106773_tag_TG_b4 |
| 8_134053240_rs11535853_non.syn_TG_b4 |
| 8_134054720_rs7015412_tag_TG_b4 |
| 8_134062846_rs3892189_tag_TG_b4 |
| 8_134177081_rs3779955_tag_TG_b13 |
| 8_134177635_rs2069568_ESE_TG_b13 |
| 8_134191878_rs2979033_tag_TG_b15 |
| 8_134192567_rs4736435_tag_TG_b15 |
| 8_134194096_rs2958681_tag_TG_b15 |
| 8_134194211_rs2979040_tag_TG_b15 |
| 8_134194864_rs1133076_non.syn_TG_b15 |
| 8_134199608_rs10096431_tag_TG_b15 |
| 8_134209728_rs17705719_tag_TG_b17 |
| 8_134212816_rs2979051_tag_TG_b17 |
| 8_134213092_rs17633144_tag_TG_b17 |
| 8_134213295_rs2294024_ESE_TG_b17 |
| 8_134223056_rs10755938_tag_TG_b17 |
| 9_4965442_rs4742060_tag_JAK2_b1 |
| 9_4969730_rs1887427_tag_JAK2_b2 |
| 9_4971602_rs10758669_tag_JAK2_b2 |
| 9_4973311_rs3808850_tag_JAK2_b2 |
| 9_4988639_rs2225125_tag_JAK2_b3 |
| 9_4993338_rs4372063_tag_JAK2_b3 |
| 9_5000192_rs10815144_tag_JAK2_b3 |
| 9_5005732_rs7046736_tag_JAK2_b3 |
| 9_5006145_rs10815147_TFBS_JAK2_b3 |
| 9_5011514_rs7034753_TFBS_JAK2_b3 |
| 9_5045434_rs1536800_tag_JAK2_b3 |
| 9_5049440_rs2149556_tag_JAK2_b3 |
| 9_5062491_rs10974945_ESE_JAK2_b3 |
| 9_5062602_rs17490221_non.syn_JAK2_b3 |
| 9_5062846_rs10974947_tag_JAK2_b3 |
| 9_5071780_rs2230724_ESE_JAK2_b3 |
| 9_5073173_rs1410779_tag_JAK2_b3 |
| 9_97247123_rs16909865_ESE_PTCH_r1 |
| 9_97249415_rs357564_non.syn_PTCH_r2 |
| 9_97278179_rs2066836_ESE_PTCH_r3 |
| 9_99645254_rs7048394_tag_FOXE1_b1 |
| 9_99652091_rs894673_tag_FOXE1_b1 |
| 9_99653961_rs3758249_TFBS_FOXE1_b1 |
| 9_99654938_rs907577_TFBS_FOXE1_b1 |
| 9_99656842_rs3021526_tag_FOXE1_b1 |
| 9_99661939_rs874004_tag_FOXE1_b1 |
| 9_99664423_rs10119760_tag_FOXE1_b1 |
| 10_51226455_rs2072701_tag_NCOA4_b1 |
| 10_51228131_rs7076948_tag_NCOA4_b1 |
| 10_51228666_rs10994470_tag_NCOA4_b1 |
| 10_51229475_rs7904463_tag_NCOA4_b1 |
| 10_51231805_rs17178655_tag_NCOA4_b1 |
| 10_51240158_rs10740051_tag_NCOA4_b2 |
| 10_51241137_rs3813713_tag_NCOA4_b2 |
| 10_51264468_rs7350420_tag_NCOA4_b2 |
| 10_61209408_rs721992_tag_CCDC6_b1 |
| 10_61209787_rs12243559_tag_CCDC6_b1 |
| 10_61212482_rs1913512_tag_CCDC6_b1 |
| 10_61216015_rs4948364_tag_CCDC6_b1 |
| 10_61217339_rs12267686_tag_CCDC6_b1 |
| 10_61218808_rs16914105_tag_CCDC6_b1 |
| 10_61219824_rs11540401_ESE_CCDC6_b1 |
| 10_61220063_rs3802695_ESE_CCDC6_b1 |
| 10_61222698_rs1053266_non.syn_CCDC6_b2 |
| 10_61230519_rs10994021_tag_CCDC6_b2 |
| 10_61230824_rs12571632_tag_CCDC6_b2 |
| 10_61231434_rs4948242_tag_CCDC6_b2 |
| 10_61231983_rs7079829_tag_CCDC6_b2 |
| 10_61236006_rs10509112_tag_CCDC6_b2 |
| 10_61246856_rs7088886_tag_CCDC6_b3 |
| 10_61250973_rs920964_tag_CCDC6_b3 |
| 10_61251195_rs17201105_tag_CCDC6_b3 |
| 10_61255837_rs10994034_tag_CCDC6_b3 |
| 10_61270958_rs4948372_tag_CCDC6_b4 |
| 10_61273589_rs3851247_tag_CCDC6_b4 |
| 10_61282038_rs17791436_tag_CCDC6_b4 |
| 10_61284289_rs1709343_tag_CCDC6_b4 |
| 10_61284917_rs2440926_tag_CCDC6_b4 |
| 10_61286390_rs17202345_tag_CCDC6_b4 |
| 10_61286612_rs1664284_tag_CCDC6_b5 |
| 10_61286730_rs1664285_tag_CCDC6_b5 |
| 10_61293808_rs10509114_tag_CCDC6_b6 |
| 10_61293894_rs2458663_tag_CCDC6_b6 |
| 10_61294703_rs1177701_tag_CCDC6_b7 |
| 10_61299829_rs1621496_tag_CCDC6_b7 |
| 10_61300256_rs1684898_tag_CCDC6_b7 |
| 10_61301422_rs1664256_tag_CCDC6_b8 |
| 10_61301769_rs1664257_tag_CCDC6_b8 |
| 10_61302122_rs1684900_tag_CCDC6_b8 |
| 10_61302634_rs1684901_tag_CCDC6_b8 |
| 10_61314974_rs1171813_tag_CCDC6_b9 |
| 10_61321707_rs1180657_tag_CCDC6_b10 |
| 10_61325303_rs1171812_tag_CCDC6_b10 |
| 10_61326899_rs1125168_tag_CCDC6_b10 |
| 10_61327506_rs2393573_tag_CCDC6_b10 |
| 10_61330162_rs3793866_tag_CCDC6_b10 |
| 10_61331570_rs7914022_tag_CCDC6_b10 |
| 10_61338345_rs3763745_tag_CCDC6_b10 |
| 10_61338573_rs1171833_tag_CCDC6_b11 |
| 10_61339057_rs3763743_tag_CCDC6_b11 |
| 10_75341362_rs2227580_non.syn_PLAU_r1 |
| 10_75343107_rs2227564_non.syn_PLAU_r2 |
| 11_3795486_rs7946577_tag_RHOG_b1 |
| 11_3795529_rs10835147_tag_RHOG_b1 |
| 11_3803869_rs1055640_tag_RHOG_b2 |
| 11_3808016_rs7948532_tag_RHOG_b3 |
| 11_3809528_rs1451721_tag_RHOG_b3 |
| 11_3812435_rs4597058_tag_RHOG_b4 |
| 11_3813129_rs1451722_tag_RHOG_b4 |
| 11_3813244_rs1451724_tag_RHOG_b4 |
| 11_3815192_rs11030008_tag_RHOG_b4 |
| 11_3823926_rs11030043_tag_RHOG_b5 |
| 11_32417040_rs6508_non.syn_WT1_r1 |
| 11_63195978_rs3016865_ESE_RTN3_b1 |
| 11_63205701_rs11551944_non.syn_RTN3_b1 |
| 11_63211715_rs7943876_tag_RTN3_b1 |
| 11_63243962_rs542998_non.syn_RTN3_b1 |
| 11_63273401_rs17657473_tag_RTN3_b1 |
| 11_63283277_rs11551942_ESE_RTN3_b1 |
| 11_63793609_rs876064_tag_BAD_b1 |
| 11_63813658_rs12794369_TFBS_BAD_r1 |
| 11_101899234_rs17886506_non.syn_MMP7_r1 |
| 11_101903803_rs10502001_non.syn_MMP7_r2 |
| 11_130255852_rs2298566_non.syn_SNX19_r1 |
| 11_130281734_rs4414223_non.syn_SNX19_r2 |
| 11_130289964_rs3751037_non.syn_SNX19_r3 |
| 12_5998704_rs216311_non.syn_VWF_r1 |
| 12_6014245_rs216321_non.syn_VWF_r2 |
| 12_6023795_rs1063856_non.syn_VWF_r3 |
| 12_6042463_rs1800378_non.syn_VWF_r4 |
| 12_27999841_rs12227702_tag_PTHLH_b1 |
| 12_28000415_rs3910902_tag_PTHLH_b1 |
| 12_28007324_rs6245_ESE_PTHLH_b1 |
| 12_28007378_rs2796_tag_PTHLH_b1 |
| 12_28010114_rs805512_tag_PTHLH_b2 |
| 12_28010651_rs997199_tag_PTHLH_b2 |
| 12_28021839_rs11049248_tag_PTHLH_b3 |
| 12_52151122_rs11170562_tag_MAP3K12_b1 |
| 12_52152886_rs784563_tag_MAP3K12_b1 |
| 12_52159633_rs4790_tag_MAP3K12_b1 |
| 12_52160111_rs1049193_tag_MAP3K12_b1 |
| 12_52166920_rs7958457_tag_MAP3K12_b1 |
| 12_55014267_rs2371494_tag_STAT2_b1 |
| 12_55026949_rs2066807_non.syn_STAT2_b1 |
| 12_100074307_rs2279834_ESE_SLC5A8_b1 |
| 12_100080857_rs7969185_tag_SLC5A8_b1 |
| 12_100084459_rs164365_non.syn_SLC5A8_b1 |
| 12_100085094_rs164364_tag_SLC5A8_b1 |
| 12_100099783_rs2671434_tag_SLC5A8_b1 |
| 12_100111649_rs1709189_non.syn_SLC5A8_b1 |
| 12_100116776_rs11110696_tag_SLC5A8_b1 |
| 12_100118718_rs10860698_tag_SLC5A8_b2 |
| 12_100119700_rs17410881_tag_SLC5A8_b2 |
| 12_100124000_rs12580634_tag_SLC5A8_b3 |
| 12_100124452_rs2712622_tag_SLC5A8_b3 |
| 12_100126478_rs7962305_tag_SLC5A8_b3 |
| 12_100128353_rs1399236_tag_SLC5A8_b3 |
| 12_100129541_rs2625160_tag_SLC5A8_b3 |
| 12_100131430_rs350463_TFBS_SLC5A8_b3 |
| 12_100135169_rs11110700_tag_SLC5A8_b3 |
| 12_100137619_rs2712623_tag_SLC5A8_b4 |
| 12_100138534_rs2062167_tag_SLC5A8_b4 |
| 12_119901033_rs1169288_non.syn_TCF1_r1 |
| 12_119919810_rs2464196_non.syn_TCF1_r2 |
| 12_119919858_rs2464195_non.syn_TCF1_r3 |
| 12_123387366_rs2230944_non.syn_NCOR2_r1 |
| 14_80481870_rs12895801_tag_TSHR_b1 |
| 14_80484500_rs10146516_TFBS_TSHR_b1 |
| 14_80485906_rs12050279_tag_TSHR_b1 |
| 14_80601507_rs11845164_tag_TSHR_b10 |
| 14_80620641_rs2300527_tag_TSHR_b10 |
| 14_80623486_rs2284734_tag_TSHR_b10 |
| 14_80623539_rs2284735_tag_TSHR_b10 |
| 14_80629997_rs12147434_tag_TSHR_b10 |
| 14_80632751_rs2075179_ESE_TSHR_b10 |
| 14_80636833_rs2300534_tag_TSHR_b11 |
| 14_80638353_rs7143914_tag_TSHR_b11 |
| 14_80639431_rs1017141_tag_TSHR_b11 |
| 14_80640841_rs7150670_tag_TSHR_b11 |
| 14_80641412_rs7151769_tag_TSHR_b11 |
| 14_80644758_rs3783941_non.syn_TSHR_b11 |
| 14_80645172_rs2080305_tag_TSHR_b11 |
| 14_80680336_rs1991517_non.syn_TSHR_b11 |
| 14_91496879_rs4904828_tag_TRIP11_b1 |
| 14_91496952_rs7154514_tag_TRIP11_b1 |
| 14_91499257_rs4900092_tag_TRIP11_b1 |
| 14_91505340_rs12587248_tag_TRIP11_b1 |
| 14_91510819_rs1051340_non.syn_TRIP11_b1 |
| 14_91522261_rs17733414_tag_TRIP11_b1 |
| 14_91527644_rs8017623_tag_TRIP11_b1 |
| 14_91529711_rs8007661_tag_TRIP11_b1 |
| 14_91549050_rs11628699_tag_TRIP11_b1 |
| 14_91555634_rs7155279_tag_TRIP11_b1 |
| 14_91564183_rs17807271_tag_TRIP11_b1 |
| 14_91567786_rs7157056_tag_TRIP11_b2 |
| 14_91569101_rs11621328_tag_TRIP11_b2 |
| 14_91574021_rs2235978_tag_TRIP11_b2 |
| 14_91575668_rs17127898_non.syn_TRIP11_r1 |
| 15_43200964_rs175088_TFBS_DUOX1_r1 |
| 15_43205544_rs16977750_TFBS_DUOX1_r2 |
| 15_43211181_rs17595239_tag_DUOX1_b1 |
| 15_43211578_rs1706810_tag_DUOX1_b1 |
| 15_43213156_rs1648282_tag_DUOX1_b1 |
| 15_43222509_rs2292465_tag_DUOX1_b2 |
| 15_43225829_rs1648305_tag_DUOX1_b2 |
| 15_43231425_rs16939752_non.syn_DUOX1_r3 |
| 15_43231810_rs1706804_ESE_DUOX1_b3 |
| 15_43232791_rs1648304_tag_DUOX1_b3 |
| 15_43233448_rs2458236_non.syn_DUOX1_b3 |
| 15_43244641_rs1648312_ESE_DUOX1_b3 |
| 15_43244750_rs2292467_ESE_DUOX1_b3 |
| 15_43244811_rs1648311_ESE_DUOX1_b3 |
| 17_4551949_rs4790689_tag_ARRB2_b1 |
| 17_4555207_rs9895782_TFBS_ARRB2_r1 |
| 17_23720820_rs2227741_non.syn_VTN_r1 |
| 17_31215519_rs2291299_tag_CCL5_b1 |
| 17_31221413_rs4795095_tag_CCL5_b1 |
| 17_31222706_rs1065341_ESE_CCL5_b1 |
| 17_35474634_rs1568400_tag_THRA_b1 |
| 17_37709372_rs9906989_tag_STAT3_b1 |
| 17_37719436_rs1053005_tag_STAT3_b1 |
| 17_37719618_rs1053004_ESE_STAT3_b1 |
| 17_37719964_rs3744483_ESE_STAT3_b1 |
| 17_37745206_rs2306580_tag_STAT3_b2 |
| 17_37748428_rs8069645_tag_STAT3_b2 |
| 17_37764060_rs9912773_tag_STAT3_b2 |
| 17_37779799_rs12949918_tag_STAT3_b2 |
| 17_37793938_rs17883843_ESE_STAT3_b2 |
| 17_37799793_rs16967738_TFBS_STAT3_b2 |
| 17_37800443_rs17320971_TFBS_STAT3_b2 |
| 17_45503724_rs2230390_non.syn_ITGA3_r1 |
| 17_45510424_rs2230392_non.syn_ITGA3_r2 |
| 17_57384759_rs12941827_tag_THRAP1_b1 |
| 17_57402424_rs12451939_tag_THRAP1_b1 |
| 17_57403256_rs6504077_tag_THRAP1_b1 |
| 17_57490950_rs4422036_tag_THRAP1_b2 |
| 17_64014117_rs16972990_TFBS_PRKAR1A_r1 |
| 17_64017451_rs4968898_tag_PRKAR1A_b1 |
| 17_64019838_rs8080306_ESE_PRKAR1A_b1 |
| 17_64029666_rs16973011_tag_PRKAR1A_b1 |
| 17_64038281_rs1064757_ESE_PRKAR1A_b1 |
| 17_64039429_rs9925_ESE_PRKAR1A_b1 |
| 17_64045740_rs16973034_tag_PRKAR1A_b2 |
| 18_44704974_rs7229639_tag_SMAD7_b1 |
| 18_44707461_rs4939827_tag_SMAD7_b1 |
| 18_44712225_rs9946510_tag_SMAD7_b2 |
| 18_44712948_rs6507877_tag_SMAD7_b2 |
| 18_44714901_rs2337106_tag_SMAD7_b3 |
| 18_44715010_rs17186877_tag_SMAD7_b3 |
| 18_44715784_rs7238442_tag_SMAD7_b3 |
| 18_44717056_rs4939830_tag_SMAD7_b3 |
| 18_44722249_rs1873191_tag_SMAD7_b4 |
| 18_44722944_rs3764482_tag_SMAD7_b4 |
| 18_44736068_rs2337143_TFBS_SMAD7_b5 |
| 18_44736905_rs2337146_TFBS_SMAD7_b5 |
| 18_46791487_rs2156010_tag_SMAD4_b1 |
| 18_46821624_rs12968012_tag_SMAD4_b1 |
| 18_46822269_rs10502913_tag_SMAD4_b1 |
| 19_2200477_rs10407022_non.syn_AMH_r1 |
| 19_10330975_rs12720356_non.syn_TYK2_r1 |
| 20_35404692_rs6017916_tag_SRC_b1 |
| 20_35410985_rs6017944_tag_SRC_b1 |
| 20_35417842_rs16986606_tag_SRC_b2 |
| 20_35432668_rs8126089_tag_SRC_b3 |
| 20_35438751_rs6090576_tag_SRC_b4 |
| 20_35443071_rs6018199_tag_SRC_b4 |
| 20_35444271_rs911496_TFBS_SRC_b4 |
| 20_35455953_rs6018257_tag_SRC_b5 |
| 20_35462245_rs1570209_tag_SRC_b5 |
| 20_35466307_rs17785475_ESE_SRC_r1 |
| 21_33637569_rs2257167_non.syn_IFNAR1_r1 |
| 21_45120423_rs170962_tag_ITGB2_b1 |
| 21_45126170_rs440555_tag_ITGB2_b1 |
| 21_45130589_rs684_tag_ITGB2_b1 |
| 21_45136241_rs235326_tag_ITGB2_b2 |
| 21_45138385_rs235328_tag_ITGB2_b2 |
| 21_45146246_rs1041457_tag_ITGB2_b3 |
| 21_45146915_rs4607021_tag_ITGB2_b3 |
| 21_45147281_rs2026882_tag_ITGB2_b3 |
| 21_45152527_rs760462_tag_ITGB2_b4 |
| 21_45153345_rs760458_tag_ITGB2_b4 |
| 21_45153843_rs760456_tag_ITGB2_b4 |
| 21_45154168_rs2838734_tag_ITGB2_b4 |
| 21_45158230_rs3788150_tag_ITGB2_b4 |
| 21_45161993_rs3788151_tag_ITGB2_b5 |
| 21_45168854_rs2838738_tag_ITGB2_b6 |
| 22_20442794_rs3810608_tag_MAPK1_b1 |
| 22_20445004_rs6928_tag_MAPK1_b1 |
| 22_20445353_rs9340_tag_MAPK1_b1 |
| 22_20520163_rs17759796_tag_MAPK1_b1 |
| 22_20521585_rs8141815_tag_MAPK1_b1 |
| 22_20525075_rs12172554_tag_MAPK1_b1 |
| 22_20534793_rs8136867_TFBS_MAPK1_b1 |
| 22_20539139_rs9610470_tag_MAPK1_b1 |
| 22_36394596_rs713835_tag_LGALS1_b1 |
| 22_36396957_rs739139_TFBS_LGALS1_b1 |
| 22_36404380_rs9622682_tag_LGALS1_b1 |
| 22_39877954_rs20551_non.syn_EP300_r1 |
| 22_39904329_rs1046088_non.syn_EP300_r2 |
| X_15269371_rs5935956_tag_FIGF_b1 |
| X_15291030_rs6629030_tag_FIGF_b1 |
| X_15291342_rs12011065_ESE_FIGF_b1 |
| X_15312319_rs6632528_tag_FIGF_b2 |
| X_15313272_rs5935959_tag_FIGF_b2 |
| X_15313948_rs5980152_tag_FIGF_b2 |
| X_15316982_rs2317327_tag_FIGF_b2 |
| X_15317563_rs2027802_tag_FIGF_b2 |
| X_15319641_rs4830943_TFBS_FIGF_b2 |
| X_15323204_rs2071182_tag_FIGF_b2 |
| X_15323550_rs2071177_tag_FIGF_b2 |
| XY_1427370_rs17886756_non.syn_IL3RA_r1 |
| XY_1457644_rs17883366_non.syn_IL3RA_r2 |

SNPs are sorted by chromosome position and gene. Each SNP is listed in the following format: “chromosome_coordinate_SNP rs_function_gene_LD block or recombination area”. Abbreviations: tag=tagSNP; TFBS=Transcription Factor Binding Site; ESE= Exonic Splicing Enhancer; non.syn=non synonymous.
