# Supplementary material for: An Epistatic Interaction between the PAX8 and STK17B Genes in Papillary Thyroid Cancer Susceptibility
Source: PLoS One. 2013 Sep 23;8(9):e74765. doi: 10.1371/journal.pone.0074765 (PMC3781145; doi:10.1371/journal.pone.0074765)
Supplement: Methods S1 — Brief description of the methods used to detect epistatic interactions. (DOC) [file pone.0074765.s004.doc]

**Supplementary methods**.

**Epistasis study**

This study consisted of two parts: (1) detection of SNP-SNP interactions in the discovery stage and (2) replication of these interactions in independent case-control series.

**Detection of SNP-SNP interactions**

Five methods were used to detect epistasis in the discovery stage. Interactions detected by at least three methods were selected for stage 2 (replication).

- **Multifactor Dimensionality Reduction (MDR).**[**REFS: 17.** Hahn LW, Ritchie MD, Moore JH (2003) Multifactor dimensionality reduction software for detecting gene-gene and gene-environment interactions. Bioinformatics 19: 376-382. **y 18.** Ritchie MD, Hahn LW, Roodi N, Bailey LR, Dupont WD, et al. (2001) Multifactor-dimensionality reduction reveals high-order interactions among estrogen-metabolism genes in sporadic breast cancer. Am J Hum Genet 69: 138-147.]

The aim of MDR is to assign each of the nine possible genotype combinations for two SNPs to two classes (high and low risk) and to select the pair of SNPs that best separates the cases and controls, so that cases tend to be classified as high risk and controls as low risk.

MDR uses 10-fold cross validation; the data is partitioned into ten subsets and a training set (with 9/10 of the subjects) and a test set (1/10 of the subjects) are selected. All possible pairs of SNPs are tested. For each SNP pair, MDR assigns the nine possible genotype combinations into the classes “high risk” and “low risk” based on the ratio of cases to controls. Next, genotype combinations within each class are grouped and the misclassification error (*i.e.* the proportion of samples misclassified as high or low risk) is obtained. The SNP pair with the smallest misclassification error is selected. Subsequently, for the selected SNP pair, the prediction error is estimated using the test set. This process is repeated ten times, for all the possible training and test sets that can be generated with the original partition into ten subsets; the consistency of the model is calculated as the proportion of times the same pair of SNPs is selected.

- **Maximum Entropy Conditional Probability Modelling (MECPM).**[REF: 19. Miller DJ, Zhang Y, Yu G, Liu Y, Chen L, et al. (2009) An algorithm for learning maximum entropy probability models of disease risk that efficiently searches and sparingly encodes multilocus genomic interactions. Bioinformatics 25: 2478-2485.]

MECPM is a method based on information theory that identifies some SNPs and SNP groups (interactions) that are used to generate a predictive model.

MECPM begins with a model which maximizes the entropy without applying any constraints, which means assigning the same probability for cases and controls for any given genotype combination. The algorithm continues searching and adding constraints. Each constraint adds information about the probability of being case (or control) given a particular genotype for a particular SNP or group of SNPs. Constraints with multiple SNPs specify interactions. Once a new constraint is chosen (see below), it is included into the model with the previous constraints, and MECPM obtains a mass probability function that applies the constraints and maximizes the entropy.

To choose new constraints, a greedy algorithm is used. This algorithm tests all possible 1-SNP and 2-SNP constraints by measuring the Kullback-Leibler divergence between two probability mass functions: one assuming the previous model and the other adding the current constraint to the previous model. The constraint with the largest Kullback-Leibler divergence is retained. The Bayesian Information Criterion (BIC) is used to avoid over fitting in the construction of the model (up to 5 SNPs). In this study, only 2-way interactions obtained by MECPM with a BIC value lower than 1.1 times the lowest BIC value were selected for further analysis.

- **SNPHarvester**[REF: 20. Yang C, He Z, Wan X, Yang Q, Xue H, et al. (2009) SNPHarvester: a filtering-based approach for detecting epistatic interactions in genome-wide association studies. Bioinformatics 25: 504-511.]

SNPHarvester starts by selecting a random pair of SNPs. A χ2 p-value is used to measure the association between the pair of SNPs and the phenotype. The algorithm iteratively swaps one of the selected SNPs (one SNP at a time) with an unselected SNP, but only if the replacement improves the χ2 p-value of the pair. These replacements continue until it is not possible to swap any SNP to improve the χ2 p-value of the pair. This method therefore detects a local optimum, and could therefore fail to detect the best interacting pair of SNPs, especially if both SNPs do not interact with other SNPs. Finally, in order to identify more than one interacting SNP pair (or group), the process is repeated until no significant interactions are identified in 20 iterations. We selected interactions with a p-value < 0.00005.

- **MegaSNPHunter**[REF: 21. Wan X, Yang C, Yang Q, Xue H, Tang NL, et al. (2009) MegaSNPHunter: a learning approach to detect disease predisposition SNPs and high level interactions in genome wide association study. BMC Bioinformatics 10: 13.]

MegaSNPHunter trains a tree boosting classifier, based on other weaker classifiers (classification and regression trees, CART). Once the CART are generated, the interactions are recovered since each path from a root to a leaf is equivalent to a group of SNPs that jointly affects the phenotype. Finally, two heuristics are used to measure the contribution to the classification, one for individual SNPs and the other to detect SNP-SNP interactions with non-additive effects, and to rank the SNPs and the interactions. We used a window length equal to the number of SNPs in our study (based on the low number of SNPs selected under our candidate gene approach).Interactions with an associated heuristic value higher than 0.5 were selected for further analysis.

- **MB-MDR**[REF: 22. Calle ML, Urrea V, Vellalta G, Malats N, Steen KV (2008) Improving strategies for detecting genetic patterns of disease susceptibility in association studies. Stat Med 27: 6532-6546.]

MB-MDR applies the following steps for each possible pair of SNPs:

Step 1: Each of the nine possible genotype combinations is assigned to a category with three values: high risk (H), low risk (L) and no evidence (0). A logistic regression approach is used to test the null hypothesis ORg=1, where the ORg is the odds ratio for subjects with genotype combination g versus all others. Genotype combinations with p<0.1 were assigned to high risk (ORg>1) or low risk (ORg<1) categories, and all others were assigned to the no evidence class.

Step 2: For each risk category H and L, a new logistic regression is carried out, obtaining an OR_H_ (odds ratio for the high-risk category versus all others) and OR_L_ (odds ratio for the low-risk category versus all others).

Step 3: The value of both Wald statistics ((beta/sd(beta))^2) from the previous logistic regressions were used to assess statistical significance, using as reference a null distribution generated by permutations of case-control status.

Both logistic regression models in the first two steps were adjusted for main effects for the two SNPs considered and potential confounders (sex, age and origin), allowing us to exclude pairs of SNPs associated only by their log-additive effects.

Interactions with a p-value lower than 0.001 were considered for further analysis.

**Replication of SNP-SNP interactions**

We added the following two new functionalities to the MB-MDR version available from http://bioinfo.cnio.es/people/cboullosa/validation/mbmdr.tar.gz:

1) Since our goal was to replicate the SNP-SNP interactions observed in the discovery stage in a second independent case-control series, genotype combinations were assigned to the categories H, L and 0, determined for series I; that is, we tested the same high- and low-risk genotype combinations in the second population. Statistical significance was tested by permuting case-control status to generate a null distribution for the Wald statistic.

2) When both categories (high and low risk) were significant (ie. the two logistic regressions were significant) in the discovery series, the complete model was tested in the validation series, using a logistic regression with a new predictive independent variable with values -1, 0 and 1 assigned to samples in the L, 0 and H categories, respectively. Thus, the complete model (and not only the high- or low-risk genotype combinations) was tested in the replication series.
